# Score equivalence of paper-, tablet-, and interactive voice response system-based versions of PROMIS, PRO-CTCAE, and numerical rating scales among cancer patients

**DOI:** 10.1186/s41687-021-00368-0

**Published:** 2021-09-17

**Authors:** Minji K. Lee, Timothy J. Beebe, Kathleen J. Yost, David T. Eton, Paul J. Novotny, Amylou C. Dueck, Marlene Frost, Jeff A. Sloan

**Affiliations:** 1 Department of Quantitative Health Sciences, Mayo Clinic, 200 First St SW, Rochester, MN 55905 USA; 2 Division of Health Policy and Management, University of Minnesota School of Public Health, 625 Michigan Ave, 27th Floor, Chicago, IL 60611 USA; 3 Department of Quantitative Health Sciences, Mayo Clinic, 13400 E. Shea Blvd., Scottsdale, AZ 85259 USA

**Keywords:** Mode effect, Mode of administration, Patient-reported outcomes, PRO-CTCAE, PROMIS, Numerical rating scale, Paper, Interactive voice response, Tablet computer, Differential item functioning

## Abstract

**Background:**

The study tests the effects of data collection modes on patient responses associated with the multi-item measures such as Patient-Reported Outcomes Measurement System (PROMIS^®^), and single-item measures such as Patient-Reported Outcomes version of the Common Terminology Criteria for Adverse Events (PRO-CTCAE), and Numerical Rating Scale (NRS) measures.

**Methods:**

Adult cancer patients were recruited from five cancer centers and administered measures of anxiety, depression, fatigue, sleep disturbance, pain intensity, pain interference, ability to participate in social roles and activities, global mental and physical health, and physical function. Patients were randomized to complete the measures on paper (595), interactive voice response (IVR, 596) system, or tablet computer (589). We evaluated differential item functioning (DIF) by method of data collection using the R software package, lordif. For constructs that showed no DIF, we concluded equivalence across modes if the equivalence margin, defined as ± 0.20 × pooled SD, completely surrounds 95% confidence intervals (CI's) for difference in mean score. If the 95% CI fell totally outside the equivalence margin, we concluded systematic score difference by modes. If the 95% CI partly overlaps the equivalence margin, we concluded neither equivalence nor difference.

**Results:**

For all constructs, no DIF of any kind was found for the three modes. The scores on paper and tablet were more comparable than between IVR and other modes but none of the 95% CI’s were completely outside the equivalence margins, in which we established neither equivalence nor difference. Percentages of missing values were comparable for paper and tablet modes. Percentages of missing values were higher for IVR (2.3% to 6.5% depending on measures) compared to paper and tablet modes (0.7% to 3.3% depending on measures and modes), which was attributed to random technical difficulties experienced in some centers.

**Conclusion:**

Across all mode comparisons, there were some measures with CI’s not completely contained within the margin of small effect. Two visual modes agreed more than visual-auditory pairs. IVR may induce differences in scores unrelated to constructs being measured in comparison with paper and tablet. The users of the surveys should consider using IVR only when paper and computer administration is not feasible.

## Background

Capturing patients’ perspectives of quality of life (QOL) effectively and efficiently is critical to designing and evaluating interventions to ameliorate the impact of cancer and its treatments. Patient-reported outcomes (PROs) provide a unique method of collecting these patient perspectives directly from the patient and without interpretation by health care providers or others. One of the issues being addressed in the PRO literature is whether the assumption that items are related to the construct in identical ways for all individuals when an instrument originally developed and used for a certain mode is modified for other modes of administration. A common method is paper and pencil self-administered questionnaire (PSAQ), in which the respondent marks responses on a paper questionnaire. Computerized self-administered questionnaire (CSAQ) is a method of data collection in which the respondent uses a computer (or mobile device) to complete a questionnaire. Interactive voice recording (IVR) system, an automated telephone system navigates the respondent through the questionnaire with recording of the questions and response options,—an alternative to computer-based data collection that allows a computer to detect voice and/or keypad inputs via telephone—brings about a myriad of other potential virtues such as convenience, affordability, reliability, and clinically feasibility. There have been recommendations to administer PROs electronically when possible in adult oncology [1], because it enables a comprehensive process for screening, feedback system with scores available to patients and/or providers in a timely fashion, service provision, and data management [1–3].

Recent studies, systematic reviews, and meta-analyses evaluating the equivalence of paper- versus computer-based electronic administration of PRO measures have found evidence of equivalence between the two [4–10]. However, most of these authors indicated that their findings could not be generalized to all forms of electronic PRO administration and all called for further testing of how PROs vary across data collection modes using randomized comparability trials.

Some studies evaluated the equivalence of the visual formats associated with paper- and computer screen-based administration and aural formats such as IVR [11–13]: With 112 patients answering 28 Patient-Reported Outcomes version of the Common Terminology Criteria for Adverse Events (PRO-CTCAE) items in three formats (i.e., paper-, computer screen-, and IVR administration), Bennett, Dueck, Mitchell et al. [11] showed moderate to high equivalence among modes using randomized crossover design, in which each participant answered the same questionnaires with more than one mode. One limitation of their study is that the screen-based or IVR questionnaires incorporated conditional branching or skip patterns that paper mode did not, which may induce mode-specific response style or non-response. Lundy, Coons, Flood et al. [13] concluded mode equivalence for paper, handheld, tablet, IVR, and web for EQ-5D-5L, in which each participant answered the questionnaire using three modes. The order of the modes was varied among participants. However, there is possibility that participants recalled their responses to the previous set of questions answered with different modes. Bjorner, Rose, Gandek, et al. [12] used a randomized crossover design where 923 adults answered parallel Patient reported Outcomes Measurement Information System (PROMIS^®^) static forms in fatigue, depression, and physical function using IVR, paper, personal digital assistant, or personal computer. They supported lack of differential item functioning in three PROMIS domains using multigroup confirmatory factor analysis and item response theory (IRT) as well as lack of clinically significant score differences across modes.

In the clinical realm, IVR has a host of features such as ease of access, increased perceived anonymity and privacy, and greater researcher control than other modes [14]. The principal downside to the use of IVR in clinical settings is that researchers cannot assume that an instrument that has been shown to have intended dimensionality, reliability, responsiveness, or interpretability using visual format has same qualities in IVRS [14]. Weiler et al. [15] found that, while there were no differences in the amount of symptoms recorded by IVR versus paper versions of allergic rhinitis response diaries, there were more missing data with the IVR, and patients overwhelmingly preferred to enter their data via paper-and-pencil. In comparing three versions of the CAHPS survey (standard print, illustration-enhanced, and telephone IVR), Shea et al. [16] found that administration times were shorter for IVR among individuals with low literacy levels. However, longer administration times were seen for IVR relative to its paper counterparts for Spanish speakers with high literacy levels, while the completion times were similar across modes for English speakers with high literacy levels.

One must be mindful of the possible effects of switching administration modes and plan to formally evaluate the effects of switching modes on non-response (both scale-level and item-level) and measurement error. To our knowledge, there have not been studies investigating the mode effects for numerical rating scales (NRS). In addition, the current study systematically tests the effect of three data collection methods (i.e., PSAQ, CSAQ which is tablet in this study, and IVR) on patient responses and potential measurement error associated with the PROMIS, PRO-CTCAE, and NRS within a variety of domains such as global health, physical function, social function, anxiety, depression, fatigue, sleep disturbance, and pain. The current study uses the randomized parallel groups design, in which each participant sees or hears each question only once because each patient answers the questionnaires using only one mode. This design overcomes possible memory effect. With regard to forced response vs. allowing patients to skip items, in many applications of electronic data capture, missing is not allowed. In PSAQ, respondents cannot be forced to respond to every item. In order to minimize features not intrinsic to modes, we opted to forego forced response in the IVR and CSAQ and allowed patients' nonresponse at the item level for all modes. Allowing research participants to skip questions they don’t wish to answer is also consistent with our IRB’s position on questionnaire-based research.

## Methods

### Sample

This study is a part of a larger study whose primary aim was to assess the convergent validity of PROMIS, PRO-CTCAE, and NRS by comparing item responses for two groups based on ECOG PS (0–1 vs. 2–4). A secondary analysis was to assess the relationship between survival status and PRO scores. In order to achieve power for all primary and secondary analyses, the sample size for the primary study was based on a superiority analysis of survival between high and low PRO score groups. In the current equivalence study comparing modes of administration, we claim equivalence when the confidence interval of the difference in outcomes between modes is within a predetermined equivalence margin that represents a clinically acceptable range of difference. Using σ of 2 based on the normative data on overall QOL NRS that include cancer trial patients [17], Δ of 0.45 corresponding to an effect size of 0.225, which may start to be considered non-negligible on a 0–10 scale, 2-sided type I error level of 5%, and the sample size of 1184, we obtain 94% statistical power. Using Δ of 0.40 corresponding to an effect size of 0.20, we obtain 86% statistical power.

There were five participating sites (Mayo Clinic, M.D. Anderson, Memorial-Sloan-Kettering, Northwestern University, and University of North Carolina). Patients with a diagnosis of cancer who were initiating active anti-cancer treatment within the next seven days, were currently receiving anti-cancer treatment, or underwent surgery for cancer treatment in the past 14 days, were recruited in-person by research study associates/data managers when arriving at a participating institution for a cancer-related appointment. Patients were accrued from the main hospital sites and satellite clinics for each institution. Eligibility criteria included adults who possess the ability to use and understand the informed consent and privacy protection documentation (written in English) and interact with the data collection modes (i.e., read and answer questions on a computer screen, listen to questions and respond using an IVR telephone system, or fill out a paper questionnaire). Each eligible patient provided informed consent. Enrollment and distribution of accrual across disease groups and institutions were facilitated by a recruitment coordinator.

The resulting sample were randomized to PSAQ (*n* = 604), CSAQ (*n* = 603), or IVR (*n* = 602). Participants were asked to complete the questionnaires while at the clinic for their visit. A study coordinator handed the participant a folded paper questionnaire booklet (PSAQ arm), an iPad tablet computer (CSAQ arm) or directed the patient to a landline (i.e., hardwired to a telephone jack) telephone with a keypad (IVR arm). Twenty-eight patients across three arms did not respond at all, and one person switched from IVR to PSAQ. Excluding these patients, we analyzed the remaining 595 patients who received paper, 596 IVR, and 589 CSAQ.

### Measures

We used PROMIS short forms and analogous NRS and PRO-CTCAE single-item rating scales. The PROMIS domains included in the study were emotional distress-anxiety, emotional distress-depression, fatigue, pain interference, pain intensity, physical function, satisfaction with social roles, sleep disturbance, global mental health, and global physical health. We administered nine version 1.0 short forms derived from PROMIS item banks: Anxiety 8a, Depression 8a, Fatigue 7a with two added items from another fatigue short form, Sleep Disturbance 8a, Pain Intensity 3a, Pain Interference 8a, Ability to participate in Social Roles and Activities 8a, Global Mental Health, Global Physical Health, and Physical Function 10a. The PROMIS scores are on T-score scale, which we used for comparing the average scores between modes, and we did not transform the T-scores to 0–100 scale.

National Cancer Institute (NCI)’s PRO-CTCAE is a pool of adverse symptom items for patient self-reporting in NCI-sponsored clinical trials. The CTCAE is an existing lexicon of clinician-reported adverse event items required for use in all NCI-sponsored trials. Patient versions of CTCAE symptom items is intended to provide clinicians with more comprehensive information about the patient experience with treatment when trials are completed and reported. The PRO-CTCAE item bank consists of five “types” of items (present/not present, frequency, severity, interference with usual or daily activities, and amount of symptom). In this study, we included frequency, severity and interference items for PRO-CTCAE. The response options were never, rarely, occasionally, frequently, and almost constantly for frequency; none, mild, moderate, severe, and very severe for severity; and not at all, a little bit, somewhat, quite a bit, and very much for interference items.

We used NRS items for overall health-related QOL, five major QOL domains (e.g., sleep, pain, anxiety, depression, and fatigue), and items for each domain for which a PROMIs measure exists (e.g., social and physical function). NRS scores and PRO-CTCAE scores are on 0–10 and 1–5 integer rating scales respectively. For comparing mean scores, NRS and PRO-CTCAE item scores were linearly transformed to 0–100 scales with higher scores indicating more of the construct in question (e.g., more fatigue, better physical function). Respondents answered 62 PROMIS items, 16 PRO-CTCAE items, and 11 NRS items.

Health literacy was measured by an item, “how confident are you filling out medical form by yourself?” Based on the findings by Chew et al. [18] of the screening threshold that optimizes both sensitivity and specificity, “Extremely” and “quite a bit” were coded as having adequate health literacy, and “somewhat”, “a little bit”, and “not at all” were coded as not having adequate health literacy.

### Measurement equivalence or lack of differential item functioning

A critical step before using instruments to compare scores from different modes of administration is determining whether items have the same meaning to members of different groups [19]. Psychometric concern for measurement equivalence arises whenever group comparisons on observable scores are the focus of research [20]. Once measurement equivalence between modes of administration has been established, quantitative cross-mode comparisons can be meaningfully conducted.

The R software package, lordif [21], was used to evaluate differential item functioning (DIF) in each of the PROMIS scales. Item-level data for all three types of measures (i.e., PROMIS, PRO-CTCAE, and NRS) were entered for each construct tested. Lordif assesses DIF using a hybrid of ordinal logistic regression and IRT framework. The main objective of fitting an IRT model under lordif is to obtain IRT trait estimates to serve as matching criterion. We tested whether the combined item set is unidimensional by conducting confirmatory factor analyses (CFA) treating the items as ordinal and using WLSMV estimator with lavaan R package [22]. Model fit was evaluated based on the Comparative Fit Index (CFI ≥ 0.95 very good fit) and the Standardized Root Mean Square Error Residual (SRMR ≤ 0.08) [23]. We also estimated the proportion of total variance attributable to a general factor (i.e., coefficient omega, ω_h_) [24]: Values of 0.70 or higher suggest that the item set is sufficiently unidimensional for most analytic procedures that assume unidimensionality [25] McFadden pseudo *R*^*2*^ change criterion of ≥ 0.02 was used to flag items for DIF [26]. A value of pseudo *R*^*2*^ less than 0.02 indicates a lack of evidence of differential interpretation of an item across modes.

### Comparison of means by modes of administration

If no DIF is found, then we can meaningfully interpret the differences in scores between modes. If differences in average scores between modes are observed, then we can conclude that these differences come purely from the characteristics of the modes rather than DIF. If DIF is found for a certain mode in a given domain, we would score patients using newly derived item parameters for that mode before conducting mean comparisons between modes.

We compared the percentages of missing values among modes using the equality of proportion test with χ^2^ test statistic to ensure missing values are not driving differences across the modes. For constructs where lack of DIF was established, we concluded equivalence across modes if the margin of small effect size, defined as ± 0.20 × pooled SD, completely surrounds 95% confidence intervals for difference in mean score. Here, one fifth of the pooled standard deviation indicates a small difference, following the observation by Coons et al. [27] that a “small” effect size difference is between 0.20 SD and 0.49 SD and that these values indicate minimal difference worthy of attention. If the 95% CI fell completely outside the margin, we concluded systematic score difference by modes. If the 95% CI partly overlaps the equivalence margin, we concluded neither equivalence nor difference.

## Results

### Sample

Patient characteristics by mode were similar, confirming successful randomization (Table 1). There were no significant differences in demographic characteristics among three modes: Distributions of age, proportions of male and female, proportion of non-white race, proportions of four different categories of education, being married, being employed or on sick leave, and having adequate health literacy had no statistically significant difference among modes of administration. In terms of medical characteristics, proportions of different types of cancers did not differ between modes as well as disease stage, ECOG performance score, types of cancer treatment in the past two weeks, and current treatment intention (i.e., curative or palliative). The only statistically significant difference in patient characteristics was found in proportion of Hispanic: 4% in PSAQ, 8% in CSAQ, and 5% in IVR arm.

**Table 1:** Patient characteristics by mode of administration

Characteristic	PSAQ	CSAQ	IVR	*p* value
Sample size	595	596	589	
*Age (in years), mean (standard deviation)*	57 (12)	56 (13)	56 (13)	.83
*Gender, female, %*	62	60	63	.52
*Race, Non-White, %*	27	24	26	.54
*Ethnicity, Hispanic, %*	4	8	5	.03
*Site, %*				.71
MD Anderson	19	19	19	
Mayo Clinic	47	48	46	
Memorial-Sloan Kettering	8	8	8	
Northwestern University	23	24	24	
University of North Carolina	3	1	3	
*Highest level of education, %*				.83
High School graduate or lower	29	29	28	
Vocational school degree, some college or college graduate	53	55	53	
Graduate or professional school degree	17	15	18	
Other	1	1	1	
*Marital status, married or marriage-like relationship, %*	72	68	71	.35
*Employment status, %*				.12
Employed (full or part time)	29	33	35	
On sick leave or disability	27	27	23	
Other	44	40	43	
*Disease, %*				.56
Breast	27	24	28	
Lymphoma/myeloma	18	23	22	
Prostate/bladder	1	1	1	
Lung	9	8	6	
Colorectal	10	11	9	
Head/neck/gastroesophageal	9	9	7	
Other	26	25	26	
*Disease stage, %*				.46
I	12	10	14	
II	21	22	22	
III	29	30	30	
IV	38	38	35	
*ECOG performance score, %*				.09
0–1	71	73	67	
2–4	29	27	33	
*Cancer treatment in the past 2 weeks, %*				.09
Chemotherapy	59	57	60	
Radiation	4	2	2	
Surgery	1	1	1	
Combination of above	8	12	8	
None of the above	29	27	30	
*Current treatment intention, %*				.58
Curative	70	70	72	
Palliative	30	30	28	
*Adequate confidence filling out medical forms, %*	85	81	84	.23

The percentages are percentages in each column for a given characteristic. PSAQ means paper-and-pencil self-administered questionnaire, CSAQ computerized self-administered questionnaire, and IVR questionnaire completed using interactive voice recording. Health literacy was measured by an item on confidence filling out medical form: “Extremely” and “quite a bit” were coded as having adequate confidence, and “somewhat”, “a little bit”, and “not at all” were coded as not having adequate confidence

### Differential item functioning

For the item sets combining PROMIS, NRS, and PRO-CTCAE, the CFA fit statistics based on CFI and SRMR were excellent for global physical health, global mental health, anxiety, depression, fatigue, sleep disturbance, pain intensity, pain interference, and ability to participate in social roles and activities. For physical function, CFI was 0.982 but SRMR was 0.126. For all constructs, ω_h_ values exceeded 0.70. For all constructs, no DIF of any kind was found for three modes (Table 2 and “Appendix 1”). All items in all analyses had a McFadden pseudo *R*^*2*^ change below the criterion that indicates DIF (< 0.02). Because there was no item exhibiting DIF among PSAQ, CSAQ, and IVR, we did not have to let items take different item parameter values depending on the modes. Across methods, patients interpreted items in similar ways.

**Table 2:** Differential item functioning by modes of administration for anxiety

Anxiety	McFadden pseudo *R*^*2*^ change (${R}_{2}^{2}-{R}_{1}^{2}$)	McFadden pseudo *R*^*2*^ change (${R}_{3}^{2}-{R}_{2}^{2}$)	McFadden pseudo *R*^*2*^ change (${R}_{3}^{2}-{R}_{1}^{2}$)
I felt fearful	0.0001	0.0007	0.0005
I found it hard to focus on anything other than my anxiety	0.0041	0.0051	0.0010
My worries overwhelmed me	0.0019	0.0028	0.0008
I felt uneasy	0.0007	0.0015	0.0008
I felt nervous	0.0017	0.0023	0.0006
I felt like I needed help for my anxiety	0.0015	0.0029	0.0014
I felt anxious	0.0002	0.0004	0.0001
I felt tense	0.0011	0.0015	0.0003
(NRS) describe the level of anxiety on average	0.0005	0.0005	0.0000
(PRO-CTCAE) How often did you feel anxiety?	0.0049	0.0058	0.0009
(PRO-CTCAE) What was the severity of your anxiety at the WORST?	0.0025	0.0025	0.0000
(PRO-CTCAE) How much did anxiety interfere with usual/daily activities?	0.0009	0.0017	0.0008

A base model (model 1) posits that only the trait level predicts responses. A second model (model 2) has both trait level and group as independent variables. If model 2 predicts item responses statistically significantly better than model 1 (i.e., McFadden pseudo *R*^*2*^ change (${R}_{2}^{2}-{R}_{1}^{2}$) ≥ 0.02), then there is uniform DIF. In uniform DIF, DIF has a consistent impact across trait levels. If the model that includes an interaction term between trait and group (model 3) fits significantly better than model 2 (i.e., McFadden pseudo *R*^*2*^ change (${R}_{3}^{2}-{R}_{2}^{2}$) ≥ 0.02), then the impact of DIF varies by trait level (nonuniform DIF). If model 3 fits significantly better than model 1 (i.e., McFadden pseudo *R*^*2*^ change (${R}_{3}^{2}-{R}_{1}^{2}$) ≥ 0.02), there is overall or total DIF

### Comparisons of scores based on modes

Table 3 has the summary scores for each of the domains and modes. The average PROMIS T- scores indicated that the study population was not demonstrably different from the general population in most constructs. The exception was in physical function: Physical function PROMIS scores of our sample were 0.6 SDs lower compared to the general population. The average difference scores between modes are presented along with the margins of small effect size in Table 4 for ease of comparison. The scores on PSAQ and CSAQ were the most similar in that 29 out of 37 CI’s of the mean differences were completely surrounded by the margins of small effect size, in which case we inferred equivalence. There were fewer results indicating equivalence between IVR and other modes: 14 out of 37 CI’s for CSAQ-IVR and 9 CI’s for PSAQ-IVR were within the margin of small effect sizes. For 95% CI’s that were not within the margin of small effect sizes, none of the CI’s were completely outside the margins, which was somewhat inconclusive in that it indicates neither equivalence nor difference. In some instances, the observed point estimate of outcome difference lied outside the equivalence margins, which indicated a clearer lack of equivalence. For example, the difference between CSAQ and IVR on NRS fatigue, 5.69, was outside the equivalence margin of [− 5.19, 5.19]. There were more of such differences between IVR and PSAQ in NRS anxiety, NRS depression, NRS fatigue, PRO-CTCAE anxiety items, a PRO-CTCAE item asking the frequency of feeling sadness, and a PRO-CTCAE item asking the severity of sleep difficulty. In general, those who responded on IVR reported higher function and lower symptoms compared to other modes, and this trend was most marked in IVR-PSAQ comparison.

**Table 3:** Health-related quality of life (HRQOL) scores by mode of administration

Domain questionnaire and scale	PSAQ mean (SD)	CSAQ mean (SD)	IVR mean (SD)
*Global/general health*			
PROMIS global mental	48.6 (8.2)	49.6 (8.5)	50.4 (7.9)
PROMIS global physical	43.7 (8.3)	44.0 (8.5)	44.1 (7.6)
NRS overall QOL	72.6 (20.7)	74.4 (20.2)	72.2 (21.5)
NRS emotional well-being	75.7 (21.0)	77.1 (19.4)	75.3 (20.4)
NRS mental well-being	80.2 (19.6)	81.1 (18.4)	78.8 (19.7)
*Physical function*			
PROMIS physical function	43.7 (8.3)	44.0 (8.5)	44.1 (7.6)
NRS physical well-being	69.6 (22.4)	71.2 (20.2)	71.9 (21.6)
*Social function*			
PROMIS social function	48.5 (9.6)	49.0 (9.6)	49.2 (8.8)
NRS social activity	66.0 (26.0)	68.5 (24.1)	69.6 (23.9)
*Emotional distress—anxiety*			
PROMIS anxiety	50.2 (9.8)	49.3 (9.6)	49.2 (9.2)
NRS anxiety	28.2 (26.6)	27.6 (27.3)	22.7 (24.1)
PRO-CTCAE anxiety frequency	32.2 (25.9)	29.3 (25.1)	25.0 (24.6)
PRO-CTCAE anxiety severity	27.7 (24.1)	25.0 (23.1)	22.0 (22.2)
PRO-CTCAE anxiety interference	16.9 (23.2)	15.3 (22.9)	12.2 (19.8)
*Emotional distress—depression*			
PROMIS depression	48.0 (9.1)	47.4 (8.9)	48.1 (7.8)
NRS depression	17.4 (23.1)	16.7 (23.2)	12.7 (18.7)
PRO-CTCAE cheer frequency	16.4 (22.5)	15.7 (22.8)	12.7 (19.5)
PRO-CTCAE cheer severity	14.3 (22.0)	13.0 (20.8)	10.4 (17.4)
PRO-CTCAE cheer interference	12.4 (20.9)	10.7 (20.1)	9.0 (17.1)
PRO-CTCAE sad frequency	29.4 (23.8)	27.7 (24.0)	24.3 (20.4)
PRO-CTCAE sad severity	24.1 (21.7)	23.3 (22.2)	20.0 (19.7)
PRO-CTCAE sad interference	14.8 (21.8)	13.6 (21.7)	11.4 (18.6)
*Fatigue*			
PROMIS fatigue	53.0 (9.0)	52.2 (9.4)	51.0 (8.7)
NRS fatigue	40.7 (26.0)	39.5 (27.1)	33.9 (24.6)
PRO-CTCAE fatigue severity	41.2 (23.8)	39.8 (24.5)	36.5 (23.4)
PRO-CTCAE fatigue interference	36.7 (26.8)	36.3 (27.7)	31.8 (24.8)
*Sleep*			
PROMIS sleep disturbance	50.1 (9.6)	49.2 (9.3)	49.6 (8.9)
NRS sleep overall	36.5 (25.2)	33.7 (23.5)	34.4 (23.4)
PRO-CTCAE sleep severity	33.9 (26.9)	30.2 (25.8)	28.1 (25.9)
PRO-CTCAE sleep interference	25.5 (26.1)	25.1 (26.3)	21.1 (24.1)
*Pain*			
PROMIS pain intensity	49.7 (10.6)	49.5 (11.0)	48.4 (10.8)
PROMIS pain interference	51.8 (9.9)	51.3 (10.0)	50.7 (9.5)
NRS pain frequency	29.3 (29.4)	27.8 (28.7)	26.0 (28.7)
NRS pain severity	25.9 (26.2)	25.9 (27.1)	23.7 (25.5)
PRO-CTCAE pain frequency	38.0 (30.6)	38.6 (32.0)	35.1 (31.8)
PRO-CTCAE pain severity	32.0 (26.8)	32.1 (27.9)	30.2 (28.4)
PRO-CTCAE pain interference	24.5 (27.9)	24.7 (28.7)	22.1 (26.8)

PSAQ means paper-and-pencil self-administered questionnaire, CSAQ computerized self-administered questionnaire, and IVR questionnaire completed using interactive voice recording. The PROMIS scores were on a T-score metric, whereas NRS and PRO-CTCAE items were transformed to 0–100 scale

**Table 4:** Differences in HRQOL scores and margins of small effect size by mode of administration

Domain questionnaire and scale	PSAQ-CSAQ mean diff (95% CI)	Margin of small effect size	CSAQ-IVR mean diff (95% CI)	Margin of small effect size	IVR-PSAQ Mean diff (95% CI)	Margin of small effect size
*Global/general health*						
PROMIS global mental	− **1.05 (**− **2.01, **− **0.09)**	± 1.67	− **0.77 (**− **1.71, 0.17)**	± 1.64	**1.82 (0.89, 2.75)**	± 1.62
PROMIS global physical	− 0.41 (− 1.39, 0.57)	± 1.70	− 0.03 (− 1.00, 0.94)	± 1.69	0.44 (− 0.52, 1.41)	± 1.67
NRS overall QOL	− **1.76 (**− **4.10, 0.58)**	± 4.09	**2.19 (**− **0.20, 4.58)**	± 4.17	− 0.43 (− 2.85, 1.99)	± 4.22
NRS emotional well-being	− 1.44 (− 3.75, 0.88)	± 4.05	**1.82 (**− **0.46, 4.10)**	± 3.99	− 0.38 (− 2.76, 1.99)	± 4.15
NRS mental well-being	− 0.88 (− 3.06, 1.29)	± 3.80	**2.24 (0.06, 4.42)**	± 3.81	− 1.36 (− 3.61, 0.89)	± 3.92
*Physical function*						
PROMIS physical function	− 0.39 (− 1.35, 0.58)	± 1.68	− 0.05 (− 0.98, 0.88)	± 1.61	0.43 (− 0.48, 1.35)	± 1.60
NRS Physical well-being	− 1.55 (− 3.99, 0.90)	± 4.26	− 0.72 (− 3.15, 1.70)	± 4.18	**2.27 (**− **0.27, 4.82)**	± 4.41
*Social function*						
PROMIS social function	− 0.51 (− 1.61, 0.59)	± 1.92	− 0.22 (− 1.28, 0.84)	± 1.84	0.73 (− 0.34, 1.79)	± 1.85
NRS social activity	− **2.43 (**− **5.32, 0.46)**	± 5.01	− 1.16 (− 3.95, 1.62)	± 4.80	**3.59 (0.70, 6.48)**	± 4.99
*Emotional distress—anxiety*						
PROMIS anxiety	0.77 (− 0.35, 1.88)	± 1.94	0.21 (− 0.88, 1.30)	± 1.89	− **0.98 (**− **2.08, 0.12)**	± 1.90
NRS anxiety	0.60 (− 2.51, 3.70)	± 5.40	**5.01 (2.02, 7.99)**	± 5.16	− **5.60 (**− **8.54, **− **2.66)**	± 5.08
PRO-CTCAE anxiety frequency	**2.89 (**− **0.04, 5.83)**	± 5.10	**4.36 (1.48, 7.23)**	± 4.97	− **7.25 (**− **10.2, **− **4.33)**	± 5.05
PRO-CTCAE anxiety severity	**2.66 (**− **0.05, 5.37)**	± 4.72	**3.09 (0.47, 5.72)**	± 4.53	− **5.76 (**− **8.44, **− **3.07)**	± 4.64
PRO-CTCAE anxiety interference	1.62 (− 1.03, 4.27)	± 4.62	**3.10 (0.62, 5.58)**	± 4.28	− **4.72 (**− **7.22, **− **2.22)**	± 4.32
*Emotional distress—depression*						
PROMIS depression	0.57 (− 0.47, 1.60)	± 1.80	− 0.56 (− 1.53, 0.41)	± 1.68	− 0.01 (− 0.99, 0.97)	± 1.70
NRS depression	0.75 (− 1.92, 3.42)	± 4.63	3.95 (1.52, 6.39)	± 4.21	− **4.70 (**− **7.14, **− **2.27)**	± 4.21
PRO-CTCAE cheer Frequency	0.62 (− 1.99, 3.23)	± 4.53	**3.05 (0.59, 5.51)**	± 4.25	− **3.67 (**− **6.11, **− **1.23)**	± 4.22
PRO-CTCAE cheer severity	1.31 (− 1.17, 3.78)	± 4.29	**2.66 (0.43, 4.89)**	± 3.84	− **3.97 (**− **6.27, **− **1.66)**	± 3.97
PRO-CTCAE cheer interference	1.73 (− 0.64, 4.09)	± 4.11	**1.72 (**− **0.44, 3.89)**	± 3.73	− **3.45 (**− **5.67, **− **1.23)**	± 3.83
PRO-CTCAE sad frequency	1.66 (− 1.10, 4.41)	± 4.78	**3.48 (0.88, 6.07)**	± 4.47	− **5.13 (**− **7.71, **− **2.55)**	± 4.44
PRO-CTCAE sad severity	0.78 (− 1.75, 3.30)	± 4.38	**3.35 (0.91, 5.78)**	± 4.20	− **4.12 (**− **6.53, **− **1.72)**	± 4.15
PRO-CTCAE sad interference	1.14 (− 1.36, 3.64)	± 4.34	**2.30 (**− **0.04, 4.64)**	± 4.04	− **3.44 (**− **5.79, **− **1.09)**	± 4.05
*Fatigue*						
PROMIS fatigue	0.72 (− 0.34, 1.77)	± 1.84	**1.31 (0.25, 2.36)**	± 1.82	− **2.02 (**− **3.05, **− **1.00)**	± 1.77
NRS fatigue	1.17 (− 1.89, 4.22)	± 5.32	**5.69 (2.68, 8.69)**	± 5.19	− **6.85 (**− **9.80, **− **3.91)**	± 5.07
PRO-CTCAE fatigue severity	1.38 (− 1.39, 4.15)	± 4.83	**3.34 (0.56, 6.12)**	± 4.79	− **4.72 (**− **7.46, **− **1.98)**	± 4.72
PRO-CTCAE fatigue interference	0.47 (− 2.66, 3.60)	± 5.46	**4.55 (1.50, 7.60)**	± 5.27	− **5.02 (**− **8.02, **− **2.02)**	± 5.17
*Sleep*						
PROMIS sleep disturbance	**0.90 (**− **0.19, 1.98)**	± 1.90	− 0.35 (− 1.40, 0.71)	± 1.82	− 0.55 (− 1.62, 0.53)	± 1.86
NRS sleep overall	**2.79 (**− **0.00, 5.59)**	± 4.88	− 0.64 (− 3.36, 2.08)	± 4.69	− **2.15 (**− **4.97, 0.67)**	± 4.87
PRO-CTCAE sleep severity	**3.70 (0.69, 6.72)**	± 5.26	2.11 (− 0.88, 5.09)	± 5.16	− **5.81 (**− **8.86, **− **2.76)**	± 5.28
PRO-CTCAE sleep interference	0.39 (− 2.62, 3.39)	± 5.23	**4.12 (1.20, 7.04)**	± 5.05	− **4.50 (**− **7.41, **− **1.60)**	± 5.02
*Pain*						
PROMIS pain intensity	0.16 (− 1.07, 1.40)	± 2.16	**1.05 (**− **0.20, 2.31)**	± 2.18	− **1.22 (**− **2.45, 0.02)**	± 2.14
PROMIS pain interference	0.56 (− 0.58, 1.70)	± 1.99	0.62 (− 0.51, 1.75)	± 1.95	− **1.18 (**− **2.30, **− **0.06)**	± 1.94
NRS pain frequency	1.53 (− 1.80, 4.85)	± 5.81	1.79 (− 1.53, 5.11)	± 5.74	− **3.32 (**− **6.68, 0.04)**	± 5.81
NRS pain severity	− 0.02 (− 3.07, 3.03)	± 5.33	**2.29 (**− **0.76, 5.34)**	± 5.26	− **2.27 (**− **5.26, 0.72)**	± 5.17
PRO-CTCAE pain frequency	− 0.56 (− 4.15, 3.02)	± 6.26	**3.60 (**− **0.09, 7.29)**	± 6.37	− **3.04 (**− **6.64, 0.57)**	± 6.24
PRO-CTCAE pain severity	− 0.12 (− 3.25, 3.01)	± 5.47	1.96 (− 1.30, 5.22)	± 5.63	− 1.83 (− 5.02, 1.36)	± 5.52
PRO-CTCAE pain interference	− 0.17 (− 3.41, 3.08)	± 5.66	**2.68 (**− **0.54, 5.89)**	± 5.56	− **2.51 (**− **5.67, 0.66)**	± 5.48

Results in bold text indicate where an upper or lower confidence limit exceeds the margin of small effect size. PSAQ means paper-and-pencil self-administered questionnaire, CSAQ computerized self-administered questionnaire, and IVR questionnaire completed using interactive voice recording. The PROMIS scores were on a T-score metric, whereas NRS and PRO-CTCAE items were transformed to 0–100 scale

Although IVR mode tended to elicit slightly higher patient-reported function and lower symptoms, there were some inconsistencies. For example, IVR scores on PROMIS global mental health were higher (despite small effect size), whereas for NRS, those in IVR arm reported lower QOL and mental/ emotional well-being items compared to CSAQ. We investigated whether this could be attributed to possible primacy (choosing the first option) on NRS items in IVR given the auditory nature of IVR (“Appendix 2”). Across all the single-item measures, after Bonferroni correction, there was a statistically significant primacy effect for NRS overall QOL in the IVR mode. In addition, there were two comparisons (i.e., (1) NRS emotional well-being and (2) NRS mental well-being) in which patients in the PSAQ and CSAQ were more likely to choose 10 compared to those in the IVR arm; thus, suggesting a recency effect (choosing the last option) for PSAQ and CSAQ.

**Fig. 1:**
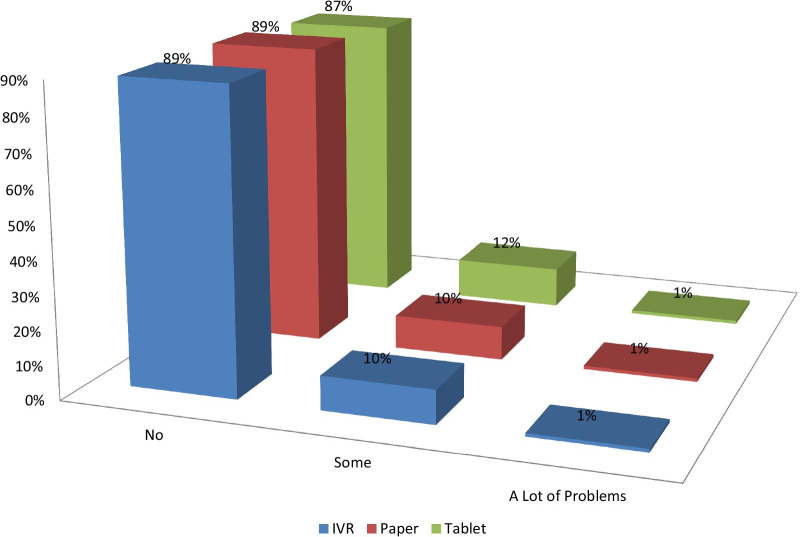
Any problems using survey today?

Percentages of missing values were comparable for PSAQ and CSAQ modes. However, percentages of missing values were slightly higher for IVR compared to PSAQ and CSAQ modes (Table 5). For multi-item scales (i.e., PROMIS short forms), we investigated whether some of the higher missing values for IVR could be due to fatigue and patients choosing not to respond to items that appear at the end of each scale. However, as “Appendix 3” shows, the percentages of missing values within each multi-item measure tended to be stable from the beginning to the end of each scale. In addition, the patients who did not respond to one scale tended to consistently not respond to other scales. Figure 1 shows that patients did not report more difficulty with IVR. All sites had more missingness from IVR than other modes. Some of these sites reported random technical problems of some data not being saved behind the scenes that patients were not aware of.

**Table 5:** Percentage of missing values in scores by mode of administration for patients with baseline data

Domain questionnaire and scale	% Missing PSAQ	% Missing CSAQ	% Missing IVR	p value all groups	p value PSAQ vs CSAQ	p value IVR vs others
*Global/general health*						
PROMIS global mental	2.8	0.7	2.3	.0201	.0050	.4151
PROMIS global physical	2.6	0.5	2.5	.0103	.0031	.0009
NRS overall QOL	1.4	0.5	2.5	.0166	.1119	.0198
NRS emotional well-being	1.4	0.3	2.5	.0068	.0482	.0119
NRS mental well-being	1.7	0.3	2.7	.0053	.0186	.0170
*Physical function*						
PROMIS physical function	1.2	1.2	3.7	.0021	.9290	.0009
NRS physical well-being	1.4	1.5	5.7	< .0001	.8513	**< .0001**
*Social function*						
PROMIS social function	1.7	1.0	5.2	< .0001	.2983	**< .0001**
NRS social activity	2.6	2.5	5.2	.0173	.9147	.0069
*Emotional distress—anxiety*						
PROMIS anxiety	2.0	1.0	5.2	< .0001	.1535	**< .0001**
NRS anxiety	2.0	1.5	5.4	.0001	.5133	**< .0001**
PRO-CTCAE anxiety frequency	2.0	1.5	5.4	.0001	.5133	**< .0001**
PRO-CTCAE anxiety severity	2.2	1.4	5.4	< .0001	.2782	**< .0001**
PRO-CTCAE anxiety interference	2.3	1.2	5.4	< .0001	.1298	**< .0001**
*Emotional distress—depression*						
PROMIS depression	2.5	1.4	4.8	.0012	.1517	.0008
NRS depression	2.8	1.9	4.8	.0109	.2812	.0068
CTC cheer frequency	2.6	1.9	4.8	.0088	.3611	.0047
CTC Cheer severity	3.3	2.0	6.2	.0006	.1822	.0004
CTC cheer interference	3.0	1.7	6.0	.0002	.1460	.0001
CTC sad frequency	3.0	1.7	6.0	.0002	.1460	.0001
CTC sad severity	2.5	1.5	6.0	< .0001	.2324	**< .0001**
CTC sad interference	2.5	1.7	6.0	< .0001	.3349	**< .0001**
*Fatigue*						
PROMIS fatigue	1.9	0.7	6.0	< .0001	.0660	**< .0001**
NRS fatigue	2.0	1.2	6.2	< .0001	.2476	**< .0001**
PRO-CTCAE fatigue severity	2.0	0.8	6.4	< .0001	.0866	**< .0001**
PRO-CTCAE fatigue interference	2.0	0.8	6.5	< .0001	.0866	**< .0001**
*Sleep*						
PROMIS sleep disturbance	0.8	0.5	6.2	< .0001	.5581	**< .0001**
NRS sleep overall	1.2	0.8	6.4	< .0001	.4976	**< .0001**
PRO-CTCAE sleep severity	1.4	0.7	5.9	< .0001	.2163	**< .0001**
PRO-CTCAE sleep interference	1.9	1.0	5.7	< .0001	.2155	**< .0001**
*Pain*						
PROMIS pain intensity	0.8	0.7	5.7	< .0001	.8393	**< .0001**
PROMIS pain interference	0.9	0.8	5.9	< .0001	.8753	**< .0001**
NRS pain frequency	1.1	0.8	5.9	< .0001	.6686	**< .0001**
NRS pain severity	0.9	1.2	5.9	< .0001	.6613	**< .0001**
PRO-CTCAE pain frequency	0.9	1.0	5.9	< .0001	.8786	**< .0001**
PRO-CTCAE pain severity	1.2	0.8	5.9	< .0001	.4976	**< .0001**
PRO-CTCAE pain interference	1.1	0.8	5.9	< .0001	.6686	**< .0001**

The equality of proportion tests with χ^2^ test statistic were used to derive the *p* values. Because of multiple comparison concerns, *p* < .0001 will be used as a guideline to identify likely significant differences

### Patient preferences for three modes

**Fig. 2:**
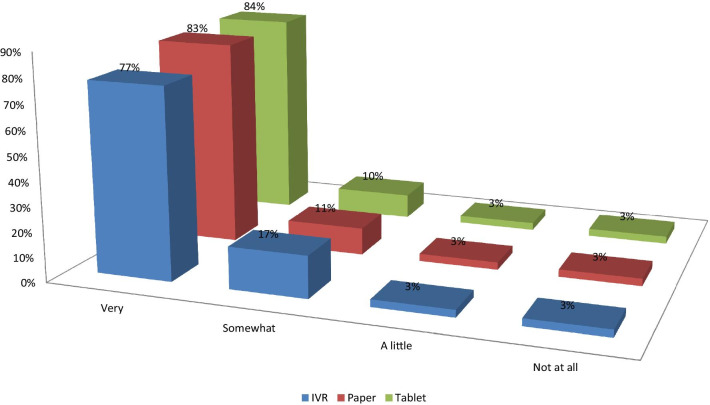
How comfortable were you when using the survey today?

**Fig. 3:**
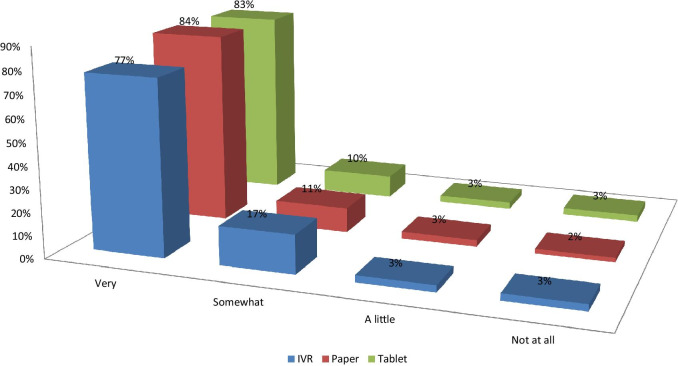
How willing to use paper survey?

**Fig. 4:**
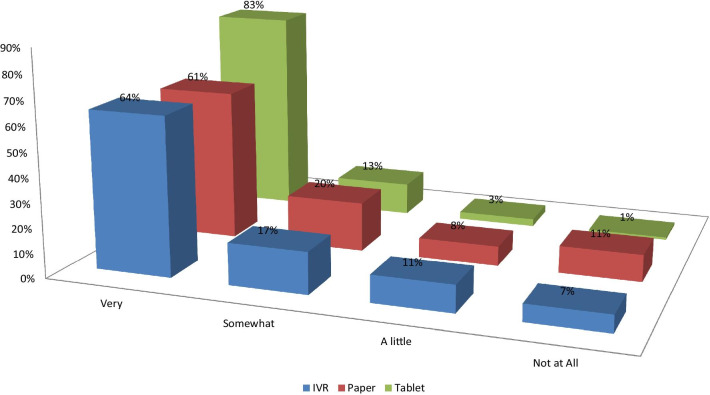
How willing to use touchscreen computer or tablet survey?

**Fig. 5:**
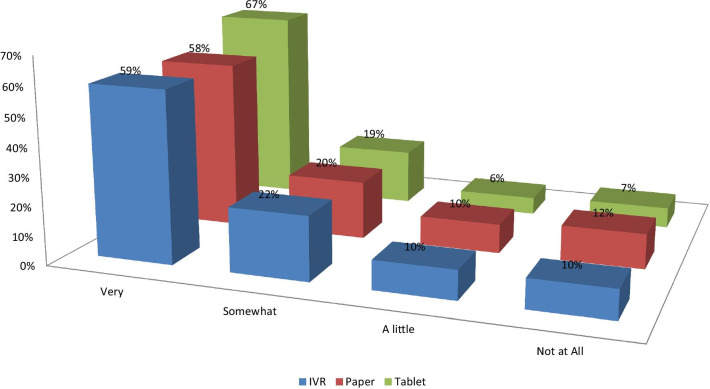
Willing to use regular computer with keyboard/mouse?

**Fig. 6:**
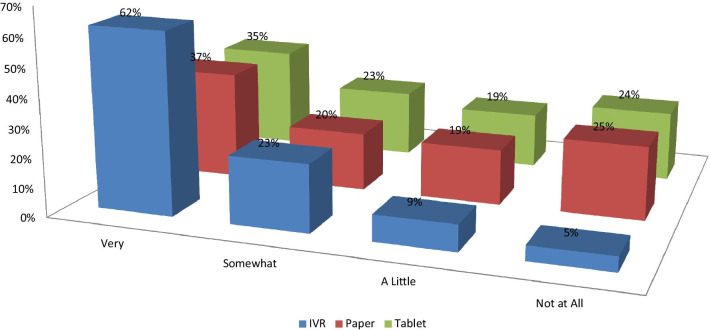
How willing to use automated telephone survey?

The proportions of patients reporting that they were very comfortable answering surveys were about 6–7% lower for IVR than PSAQ and CSAQ (Fig. 2). Patients who were randomized to PSAQ (84%) and CSAQ (83%) were more likely to say that they are very willing to use PSAQ in the future (Fig. 3); The patients who were randomized to IVR were less likely (6–7% less likely) to respond that they are very willing to use PSAQ. Patients who were randomized to CSAQ were most likely to respond that they are very willing to use touchscreen computer or tablet in the future (18–22% more willing compared to other modes) (Fig. 4). Patients randomized to CSAQ were also 8–9% more willing to use regular computers with mouse and keyboard than those randomized to other modes (Fig. 5). Lastly, patients who were randomized to IVR were most likely to respond that they were very willing to use IVR (62% compared to 37% for PSAQ and 35% for CSAQ; Fig. 6). In general, the use of a specific mode positively influenced future receptiveness to that mode.

## Discussion

The various patient demographic and medical characteristics mostly did not differ by mode. There was no DIF by mode using the lordif methods. Scores for IVR reflected higher function and lower symptoms on average than PSAQ and CSAQ for some domains and measures: When this pattern appeared, it was small effect size (e.g., 1 to 2 score point difference in PROMIS, 0.5 to 0.7 score difference in 0–10 NRS scale, and 0.17 to 0.34 score difference in 1–5 PRO-CTCAE scale). While the confidence intervals for the differences were not completely outside the equivalence margins, the straddle occurred most prominently for IVR compared with other modes in symptom domains. The highest agreement between PSAQ and CSAQ and lowest between PSAQ and IVR were also reported in the systematic review and meta-analysis of studies conducted between 2007 and 2013 [7]. Our randomized parallel groups design removes the potential practice effect, which may explain some of the non-equivalent findings between PSAQ and CSAQ that were not completely within the margin of small effect size.

Patients reporting lower symptoms and higher social or physical functions may suggest that the IVR that used recordings of a pleasant female voice in our study induced social desirability bias, where participants over-reported functional well-being and under-reported symptoms as if they were speaking with a real person. If so, this is consistent with the general survey research findings that auditory modes (e.g., IVR/ Phone) yield more positive responses than visual modes [28, 29]. However, social desirability bias for NRS could not be supported in the overall QOL and mental or emotional well-being, because IVR scores were lower on average than CSAQ. The finding that patients were less likely to choose the last option on NRS in these domains could partially explain this anomaly. It is possible that patients were less likely to press two digits to report the highest response option of 10, (i.e., one and zero), than a single digit on the keypad.

## Conclusions

Because PRO instruments may be administered in a variety of ways, it is critical for the validity of the use of the scores to know if participants would provide the same answers regardless of the modes of administration. Across all comparisons PSAQ-CSAQ, CSAQ-IVR, IVR-PSAQ and across all three kinds of measures, PROMIS, NRS, and PRO-CTCAE, there were some mean differences with CI’s upper or lower limit exceeding the margin of small effect. In the current study, the two visual modes (i.e., PSAQ vs CSAQ) agreed more than visual-auditory pairs (i.e., PSAQ vs IVR or CSAQ vs IVR). Several point estimates of score difference lying outside the margin of equivalence suggest that the IVR mode may induce some real differences in scores that are unrelated to the construct being measured, in comparison with PSAQ and CSAQ, depending on the instruments and domains. Primacy effect was supported for IVR in NRS overall QOL and PRO-CTCAE anxiety frequency items. The tendency not to choose the last option was supported for IVR in NRS emotional and mental well-being items, which may be related to participants less willing to record 10 on keypad than single-digit numbers. The next step would to be conduct cognitive interviews to understand these effects for NRS items in IVR. Although the missing data percentages were small in general, there were more missing responses using IVR compared to other modes. The limitation of the study is that we could not differentiate patients who simply did not call in or who broke off the assessment from those whose data have not been saved due to technical problems. Further research should be conducted to understand what contributes to higher missing responses in IVR. In addition, considering some sites experienced issues regarding IVR data storage, the technical aspects of IVR implementation should be checked any time large data collection is planned through this mode. In their meta-analysis, Muehlhausen et al. [7] noted that further research into standards for IVR may be needed to support the equivalence between IVR and other platforms. Because of the non-conclusive equivalence, we may not yet need to consider adjusting for method of data collection when combining data collected via IVR with PSAQ or CSAQ for these PROs. However, because of the greater amount of inconclusive results for IVR, the users of the surveys should consider using IVR only when paper and computer administration is not feasible.

## Data Availability

Data can be made available upon reasonable request to the principal investigator (J. Sloan). All requests will be reviewed.

## Appendix 1

See Table 6.

**Table 6:** Differential item functioning by modes of administration

	McFadden pseudo *R*^*2*^ change (${R}_{2}^{2}-{R}_{1}^{2}$)	McFadden pseudo *R*^*2*^ change (${R}_{3}^{2}-{R}_{2}^{2}$)	McFadden pseudo *R*^*2*^ change (${R}_{3}^{2}-{R}_{1}^{2}$)
*Anxiety*			
I felt fearful	0.0001	0.0007	0.0005
I found it hard to focus on anything other than my anxiety	0.0041	0.0051	0.0010
My worries overwhelmed me	0.0019	0.0028	0.0008
I felt uneasy	0.0007	0.0015	0.0008
I felt nervous	0.0017	0.0023	0.0006
I felt like I needed help for my anxiety	0.0015	0.0029	0.0014
I felt anxious	0.0002	0.0004	0.0001
I felt tense	0.0011	0.0015	0.0003
(NRS) Describe the level of anxiety on average	0.0005	0.0005	0.0000
(PRO-CTCAE) How often did you feel anxiety?	0.0049	0.0058	0.0009
(PRO-CTCAE) What was the severity of your anxiety at the WORST?	0.0025	0.0025	0.0000
(PRO-CTCAE) How much did anxiety interfere with usual/daily activities?	0.0009	0.0017	0.0008
*Depression*			
I felt that nothing could cheer me up	0.0144	0.0197	0.0053
I felt worthless	0.0000	0.0007	0.0007
I felt helpless	0.0011	0.0013	0.0001
I felt depressed	0.0055	0.0070	0.0015
I felt hopeless	0.0023	0.0027	0.0004
I felt like a failure	0.0004	0.0009	0.0005
I felt unhappy	0.0041	0.0046	0.0005
I felt that I had nothing to look forward to	0.0031	0.0033	0.0002
(NRS) Describe level of depression on average	0.0018	0.0020	0.0002
(PRO-CTCAE) How often did you feel nothing could cheer you up	0.0014	0.0015	0.0001
(PRO-CTCAE) What was the SEVERITY of feelings that nothing could cheer you up at the WORST?	0.0007	0.0007	0.0000
(PRO-CTCAE) How much did feeling nothing could cheer you up INTERFERE with activities?	0.0002	0.0008	0.0006
(PRO-CTCAE) How OFTEN did you have sad or unhappy feelings	0.0062	0.0066	0.0004
(PRO-CTCAE) What was the SEVERITY of your sad/unhappy feelings at the WORST?	0.0044	0.0047	0.0003
(PRO-CTCAE) How much did sad/unhappy feelings INTERFERE with activities?	0.0003	0.0003	0.0001
*Fatigue*			
How often did you feel tired?	0.0005	0.0007	0.0002
How often did you experience extreme exhaustion?	0.0002	0.0002	0.0000
How often did you run out of energy?	0.0014	0.0017	0.0003
How often did your fatigue limit you at work?	0.0001	0.0004	0.0003
How often were you too tired to think clearly?	0.0000	0.0004	0.0004
How often were you too tired to take a bath or shower?	0.0006	0.0012	0.0007
How often did you have enough energy to exercise strenuously?	0.0005	0.0013	0.0007
How often did you have to push yourself to get things done because of your fatigue?	0.0023	0.0029	0.0006
How often did you have trouble finishing things because of your fatigue?	0.0002	0.0005	0.0003
(NRS) Describe level of fatigue	0.0003	0.0010	0.0007
(PRO-CTCAE) What was severity of fatigue at its worst?	0.0000	0.0002	0.0001
(PRO-CTCAE) How much did fatigue interfere with activities?	0.0000	0.0005	0.0002
*Pain interference*			
How much did pain interfere with your family life?	0.0001	0.0002	0.0001
How much did pain interfere with work around the home?	0.0010	0.0021	0.0011
How much did pain interfere with your ability to participate in social activities?	0.0026	0.0029	0.0003
How much did pain interfere with your enjoyment of life?	0.0002	0.0004	0.0003
How much did pain interfere with the things you usually do for fun?	0.0007	0.0009	0.0002
How much did pain interfere with your enjoyment of social activities?	0.0002	0.0007	0.0005
How much did pain interfere with your household chores?	0.0003	0.0009	0.0006
(PRO-CTCAE) How much did pain interfere with activities?	0.0009	0.0019	0.0010
*Pain intensity*			
How intense was pain at its worst?	0.0016	0.0026	0.0010
How intense was your average pain?	0.0014	0.0018	0.0004
What is your level of pain right now?	0.0001	0.0002	0.0002
(NRS) Rate severity of pain	0.0000	0.0003	0.0003
(PRO-CTCAE) What was severity of pain at its worst?	0.0005	0.0015	0.0010
*Physical function*			
Does your health now limit you in doing vigorous activities such as running, lifting heavy objects, participating in strenuous sports?	0.0013	0.0018	0.0005
Are you able to get on and off the toilet?	0.0000	0.0014	0.0014
Does your health now limit you in walking more than a mile?	0.0010	0.0012	0.0002
Does your health now limit you in climbing one flight of stairs?	0.0003	0.0003	0.0000
Does your health now limit you in lifting or carrying groceries?	0.0011	0.0012	0.0001
Does your health now limit you in bending, kneeling, or stooping?	0.0009	0.0015	0.0006
Are you able to do chores such as vacuuming or yard work?	0.0020	0.0042	0.0022
Are you able to dress yourself, including tying shoelaces and doing buttons?	0.0018	0.0021	0.0003
Are you able to shampoo your hair?	0.0053	0.0061	0.0008
Are you able to wash and dry your body?	0.0021	0.0043	0.0022
(NRS) Overall physical well-being	0.0003	0.0006	0.0004
*Sleep disturbance*			
My sleep quality was	0.0013	0.0023	0.0010
My sleep was refreshing	0.0002	0.0005	0.0002
I had a problem with my sleep	0.0007	0.0011	0.0004
I had difficulty falling asleep	0.0013	0.0021	0.0008
My sleep was restless	0.0007	0.0012	0.0006
I tried hard to get to sleep	0.0002	0.0006	0.0004
I worried about not being able to fall asleep	0.0003	0.0005	0.0001
I was satisfied with my sleep	0.0001	0.0001	0.0000
(NRS) Describe quality of sleep on average	0.0002	0.0006	0.0004
(PRO-CTCAE) What was the severity of your insomnia at its worst?	0.0039	0.0045	0.0007
(PRO-CTCAE) How much has insomnia interfered with usual activities?	0.0045	0.0051	0.0006
*Ability to participate in social roles and activities*			
I have trouble doing all of my regular leisure activities with others	0.0010	0.0021	0.0011
I have trouble doing all of the family activities that I want to do	0.0005	0.0010	0.0005
I have trouble doing all of my usual work (include work at home)	0.0001	0.0009	0.0009
I have trouble doing all of the activities with friends that I want to do	0.0001	0.0006	0.0004
I have to limit the things I do for fun with others	0.0002	0.0008	0.0006
I have to limit my regular activities with friends	0.0002	0.0004	0.0001
I have to limit my regular family activities	0.0008	0.0009	0.0001
I have trouble doing all of the work that is really important to me (include work at home)	0.0013	0.0016	0.0003
(NRS) Rate level of social activity	0.0007	0.0008	0.0001
*Global mental health*			
In general, would you say your quality of life is…?	0.0008	0.0025	0.0017
In general, how would you rate your mental health, including your mood and your ability to think?	0.0074	0.0094	0.0019
In general, how would you rate your satisfaction with your social activities and relationships?	0.0002	0.0012	0.0010
How would you rate your fatigue on average?	0.0067	0.0069	0.0002
(NRS) Rate your overall QOL	0.0003	0.0005	0.0002
(NRS) Rate your emotional well-being	0.0008	0.0023	0.0015
(NRS) Rate your mental well-being	0.0035	0.0046	0.0011
*Global physical health*			
In general, how would you rate your physical health?	0.0045	0.0049	0.0004
To what extent are you able to carry out everyday physical activities such as walking, climbing stairs, carrying groceries, or moving a chair?	0.0041	0.0043	0.0001
Rate fatigue on average	0.0039	0.0041	0.0003
(NRS) Rate pain on average	0.0000	0.0001	0.0000
(ECOG) Best description of current activity level	0.0026	0.0027	0.0001

A base model (model 1) posits that only the trait level predicts responses. A second model (model 2) has both trait level and group as independent variables. If model 2 predicts item responses statistically significantly better than model 1 (i.e., McFadden pseudo *R*^*2*^ change (${R}_{2}^{2}-{R}_{1}^{2}$) ≥ 0.02), then there is uniform DIF. In uniform DIF, DIF has a consistent impact across trait levels. If the model that includes an interaction term between trait and group (model 3) fits significantly better than model 2 (i.e., McFadden pseudo *R*^*2*^ change (${R}_{3}^{2}-{R}_{2}^{2}$) ≥ 0.02), then the impact of DIF varies by trait level (nonuniform DIF). If model 3 fits significantly better than model 1 (i.e., McFadden pseudo *R*^*2*^ change (${R}_{3}^{2}-{R}_{1}^{2}$) ≥ 0.02), there is overall or total DIF

## Appendix 2

See Table 7.

**Table 7:** Percentages of selecting the first or the last response option in single-item measures

Group	PRO	Choosing the first option			Choosing the last option		
		% Paper	% Web	% IVR	% Paper	% Web	% IVR
General health	NRS overall QOL	**0.0**	**0.2**	**1.4**	18.4	18.3	13.7
	NRS emotional well-being	0.2	0.0	1.2	**22.5**	**19.6**	**14.9**
	NRS mental well-being	0.2	0.2	1.4	**30.1**	**25.7**	**19.8**
Physical function	NRS physical well-being	0.5	0.2	0.7	13.8	11.6	14.2
Social function	NRS social activity	1.4	1.2	1.2	16.4	15.2	15.7
Anxiety	NRS anxiety	25.5	28.8	30.6	0.7	0.9	0.2
	PRO-CTCAE anxiety frequency	**27.7**	**31.6**	**37.7**	1.2	1.0	1.4
	PRO-CTCAE anxiety severity	31.9	34.6	39.6	1.0	0.5	1.1
	PRO-CTCAE anxiety interference	57.7	61.0	66.4	0.7	1.5	0.4
Depression	NRS depression	47.8	46.9	51.4	0.3	0.7	0.4
	PRO-CTCAE cheer frequency	57.6	59.5	63.6	1.0	1.0	0.7
	PRO-CTCAE cheer severity	63.7	66.2	68.6	0.9	0.2	0.4
	PRO-CTCAE cheer interference	67.5	72.0	73.3	0.7	0.9	0.4
	PRO-CTCAE sad frequency	27.3	30.1	29.9	0.9	1.0	0.5
	PRO-CTCAE sad severity	33.6	35.2	38.9	0.9	1.2	1.1
	PRO-CTCAE sad interference	60.9	63.6	66.8	0.9	1.0	0.4
Fatigue	NRS fatigue	10.1	11.2	12.1	1.0	1.0	0.9
	PRO-CTCAE fatigue severity	11.3	13.0	13.2	2.9	3.1	2.7
	PRO-CTCAE fatigue interference	20.2	22.9	24.4	3.4	3.4	1.6
Sleep	NRS sleep overall	11.2	10.4	8.1	1.2	0.5	1.8
	PRO-CTCAE sleep severity	27.3	30.8	34.9	2.7	1.4	1.8
	PRO-CTCAE sleep interference	40.5	41.3	46.9	1.5	1.9	0.9
Pain	NRS pain frequency	30.2	31.3	34.5	2.9	2.1	3.6
	NRS pain severity	30.0	32.6	34.7	0.7	1.2	1.1
	PRO-CTCAE pain frequency	26.1	28.1	32.0	6.3	8.2	7.5
	PRO-CTCAE pain severity	28.1	30.3	34.3	2.9	2.9	3.0
	PRO-CTCAE pain interference	45.0	47.1	50.0	3.4	3.3	2.0

The comparisons with statistically significant differences in percentages after Bonferroni correction are bolded

## Appendix 3

See Table 8.

**Table 8:** Missing data percentage by items and modes in PROMIS items

PROMIS short forms	V1 (%)	V2 (%)	V3 (%)	V4 (%)	V5 (%)	V6 (%)	V7 (%)	V8 (%)	V9 (%)	V10 (%)
*Pain intensity*										
PSAQ	1	1	2							
IVR	6	6	6							
CSAQ	1	1	1							
Anxiety										
PSAQ	2	2	2	2	2	2	3	2	2	
IVR	5	5	6	5	6	5	6	5	5	
CSAQ	1	1	1	1	1	2	1	2	2	
*Depression*										
PSAQ	3	3	3	3	3	3	3	3		
IVR	5	5	5	5	5	5	5	5		
CSAQ	1	2	2	2	1	2	2	2		
*Fatigue*										
PSAQ	2	2	2	3	2	2	3	2	2	
IVR	6	6	6	6	7	6	6	6	6	
CSAQ	1	1	1	1	1	1	1	1	1	
*Pain interference*										
PSAQ	1	1	1	1	1	1	1			
IVR	6	6	6	6	6	6	6			
CSAQ	1	1	1	1	1	1	1			
*Sleep disturbance*										
PSAQ	2	1	1	1	1	2	2	1		
IVR	6	6	6	6	7	6	6	6		
CSAQ	1	1	1	1	1	1	1	1		
*Social roles and activities*										
PSAQ	2	2	2	2	2	2	2	2	3	
IVR	5	5	5	5	5	5	6	5	5	
CSAQ	1	1	1	1	1	1	2	2	3	
*Physical function*										
PSAQ	2	1	1	2	2	2	2	3	1	1
IVR	4	4	4	4	4	4	4	4	4	4
CSAQ	1	1	1	1	1	1	1	1	1	1

The columns from V1 through V10 indicate simply items administered by order in each scale

## Abbreviations

PROMIS: Patient-Reported Outcomes Measurement System
PRO-CTCAE: Patient-Reported Outcomes version of the Common Terminology Criteria for Adverse Events
NRS: Numerical rating scale
IVR: Interactive voice response
DIF: Differential item functioning
PRO: Patient-reported outcomes
PSAQ: Paper and pencil self-administered questionnaire
CSAQ: Computerized self-administered questionnaire
